# Supplementary material for: More Reliable EEG Electrode Digitizing Methods Can Reduce Source Estimation Uncertainty, but Current Methods Already Accurately Identify Brodmann Areas
Source: Front Neurosci. 2019 Nov 6;13:1159. doi: 10.3389/fnins.2019.01159 (PMC6856631; doi:10.3389/fnins.2019.01159)
Supplement: Supplementary file 1 [file Data_Sheet_1.PDF]

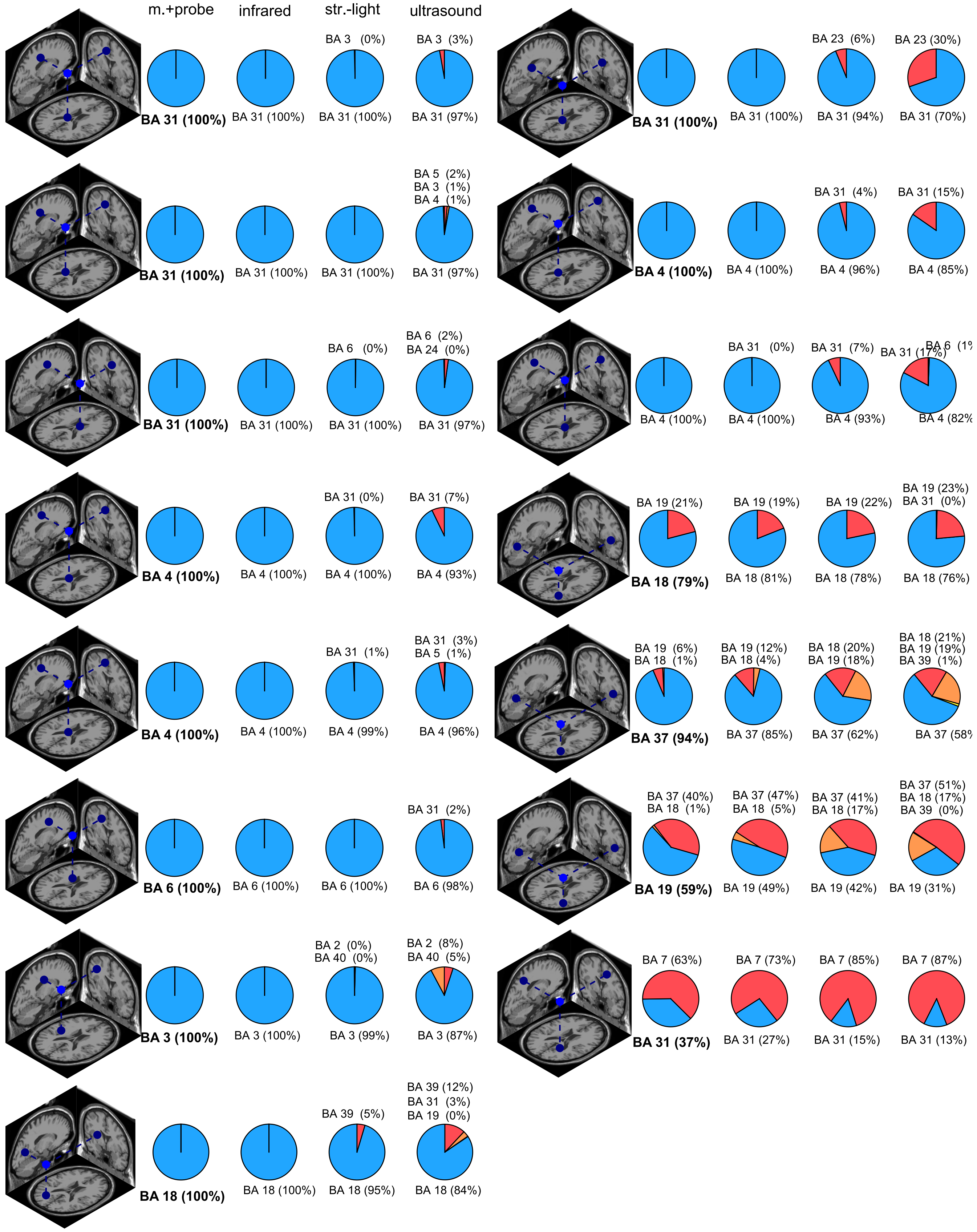

Figure S1. Brodmann area (BA) accuracy for the ICs not presented in the Figure 6.

Table S1. Manufacturer-reported accuracy and precision, calibration error, and digitization variability. Manufacturer accuracy and precision, if available, are based on the device information provided by the manufacturer. Calibration error, if available, is provided by the device calibration software. Digitization reliability is from the results of this study. str.-light 3D scan = structured-light 3D scan. mocap = motion capture. N/A = not available.

method	manufacturer accuracy & precision (cm)	calibration error (cm)	digitization reliability (cm)
ultrasound	<0.08 (precision)	<0.1	0.86
infrared 3D scan	<0.1 (precision), for scanning objects 50 cm apart	N/A	0.24
str.-light 3D scan	<0.03 (accuracy)	N/A	0.50
mocap probe	N/A	<0.04 mocap + <0.04 probe	0.15
mocap	N/A	<0.04	0.001
